# Attention-based recurrent neural network for influenza epidemic prediction

**DOI:** 10.1186/s12859-019-3131-8

**Published:** 2019-11-25

**Authors:** Xianglei Zhu, Bofeng Fu, Yaodong Yang, Yu Ma, Jianye Hao, Siqi Chen, Shuang Liu, Tiegang Li, Sen Liu, Weiming Guo, Zhenyu Liao

**Affiliations:** 1 College of Intelligence and Computing, Tianjin University, Peiyang Park Campus: No.135 Yaguan Road, Haihe Education Park, Tianjin, 300350 China; 2 Automotive Data Center, China Automotive Technology & Research, Tianjin, 300300 China; 3 Guangzhou Center for Disease Control and Prevention, Guangzhou, 510440 China; 4 Pony Testing International Group, Tianjin, 300051 China; 5 Tianjin FoodSafety Inspection Technology Institute, Tianjin, 300300 China

**Keywords:** Influenza epidemic prediction, Attention mechanism, Multi-channel LSTM neural network

## Abstract

**Background:**

Influenza is an infectious respiratory disease that can cause serious public health hazard. Due to its huge threat to the society, precise real-time forecasting of influenza outbreaks is of great value to our public.

**Results:**

In this paper, we propose a new deep neural network structure that forecasts a real-time influenza-like illness rate (ILI%) in Guangzhou, China. Long short-term memory (LSTM) neural networks is applied to precisely forecast accurateness due to the long-term attribute and diversity of influenza epidemic data. We devise a multi-channel LSTM neural network that can draw multiple information from different types of inputs. We also add attention mechanism to improve forecasting accuracy. By using this structure, we are able to deal with relationships between multiple inputs more appropriately. Our model fully consider the information in the data set, targetedly solving practical problems of the Guangzhou influenza epidemic forecasting.

**Conclusion:**

We assess the performance of our model by comparing it with different neural network structures and other state-of-the-art methods. The experimental results indicate that our model has strong competitiveness and can provide effective real-time influenza epidemic forecasting.

## Background

Influenza is an infectious respiratory disease that can cause serious public health hazard. It can aggravate the original underlying disease after infection, causing secondary bacterial pneumonia and acute exacerbation of chronic heart and lung disease. Furthermore, the 2009 H1N1 pandemic caused between 151,700 and 575,400 deaths in worldwide during the first year the virus circulated [1]. Therefore, precise on-line monitoring and forecasting of influenza epidemic outbreaks has a great value to public health departments. Influenza detection and surveillance systems provide epidemiologic information that can help public health sectors develop preventive measures and assist local medical institutions in deployment planning [2].

Influenza-like-illness (ILI) is an infectious respiratory infection measurement defined by the World Health Organization (WHO). ILI with a measured fever higher than 38^∘^C, and cough, with onset within the previous 10 days [3]. Our prediction target, ILI%, is equal to the ratio of the influenza-like cases number to the visiting patients’ number. In the field of influenza surveillance, ILI% is often used as an indicator to help determine if there is a possible influenza epidemic. When the ILI% baseline is exceeded, the influenza season has arrived, reminding the health administrations to take timely preventive measures.

In recent years, more and more researchers have concentrated on precise on-line monitoring, early detection and influenza epidemic outbreaks forecasting. Thus, influenza epidemic outbreaks forecasting has become the most active research direction. The information from website search or social network applications, such as Twitter and Google Correlate[4–6], provides sufficient data support for this research area. Previous methods are commonly built on linear models, such as least absolute shrinkage and selection operator (LASSO) or penalized regression[4, 6, 7]. Some people also implement deep learning models when solving influenza epidemic forecasting problems[8, 9]. However, these methods can’t efficiently provide the precise forecasting of ILI% one week in advance. First, the online data is not accurate enough and lacks necessary features, which cannot fully reflect the trend of the influenza epidemic. Second, influenza epidemic data is usually very complex, non-stationary, and very noisy. Traditional linear models cannot handle multi-variable inputs appropriately. Third, previously proposed deep learning methods didn’t consider the time-sequence property of influenza epidemic data.

In this paper, we use influenza surveillance data as our data set, which is provided by the Guangzhou Center for Disease Control and Prevention. This data set includes multiple features and is count separately of each district in Guangzhou. Our approach takes advantage of these two characteristics. Meanwhile, we consider the time-sequence property, making our approach solve the influenza epidemic forecasting problem in Guangzhou with pertinence. Due to the relevant specifications of data collection, our method is also applicable in other regions.

We concentrate on implementing deep learning models to address the influenza outbreaks forecasting problem. Recently, deep learning methods have obtained remarkable performances in various research areas from computer vision, speech recognition to climate forecasting[10–12]. We implement long-short term memory (LSTM) neural networks[13] as a fundamental method for forecasting, because the influenza epidemic data naturally has time series attribute. Considering that different types of input data correspond to different characteristics, one single LSTM with a specific filter may not capture the time series information comprehensively. By using a multi-channel architecture, we can better capture the time series attributes from the data. Not only ensures the integration of various relevant descriptors in the high-level network, but also ensures that the input data will not interfere with each other in the underlying network. The structured LSTM can provide robust fitting ability that has been provided in several papers [14, 15]. We further enhance our method using attention mechanism. In attention layer, the probability of occurrence of each value in the output sequence depends on the values in the input sequence. By designing this architecture, we can better deal with input stream relationships among multiple regions more appropriately. We named our model as Att-MCLSTM, which stands for attention-based multi-channel LSTM.

Our main contributions can be summarized as follows: (1) We test our model on Guangzhou influenza surveillance data set, which is authentic and reliable. It contains multiple attributes and time series features. (2) We propose an attention-based multi-channel LSTM structure that associates different well-behaved approaches. The structure takes the forecasting problem and the influenza epidemic data attributes into account. The proposed model can be seen as an alternative to forecast influenza epidemic outbreaks in other districts. (3) The proposed model makes full use of information in the data set, solving the actual problem of influenza epidemic forecasting in Guangzhou with pertinence. The experimental results demonstrate the validity of our method. To the best of our knowledge, this is the first study that applies LSTM neural networks to the influenza outbreaks forecasting problem.

The rest of this paper is organized as follows. In the second section, we illustrate details of our method. In the third section, we evaluate performances of our method by comparing it with different neural network structures and other prior art methods. In the fourth section, we discuss conclusions and prospects for future works.

## Methods

The accurateness of the forecasting problems can be enhanced by combining multiple models[16–26]. In this paper, we devise an novel LSTM neural network structure to settle the influenza epidemic forecasting problem in Guangzhou, China. Our model can extract characteristics more effectively from time series data, and take different impacts of different parts of data into consideration. In order to illustrate our model clearly, we illustrate our data set first. The following sections will give further illustrations on the data set, the overall idea of our model, details of LSTM neural networks, attention mechanism, attention-based multi-channel LSTM, data normalization, and evaluation method.

### Data set description

The influenza surveillance data we used includes 9 years data. Statistics on influenza epidemic data in 9 regions are counted each year. The data set includes 6 modules, and each of these modules has multiple features. The data set has one record each week, and data for 52 weeks is counted each year.

### Design of the proposed model

**Fig. 1:**
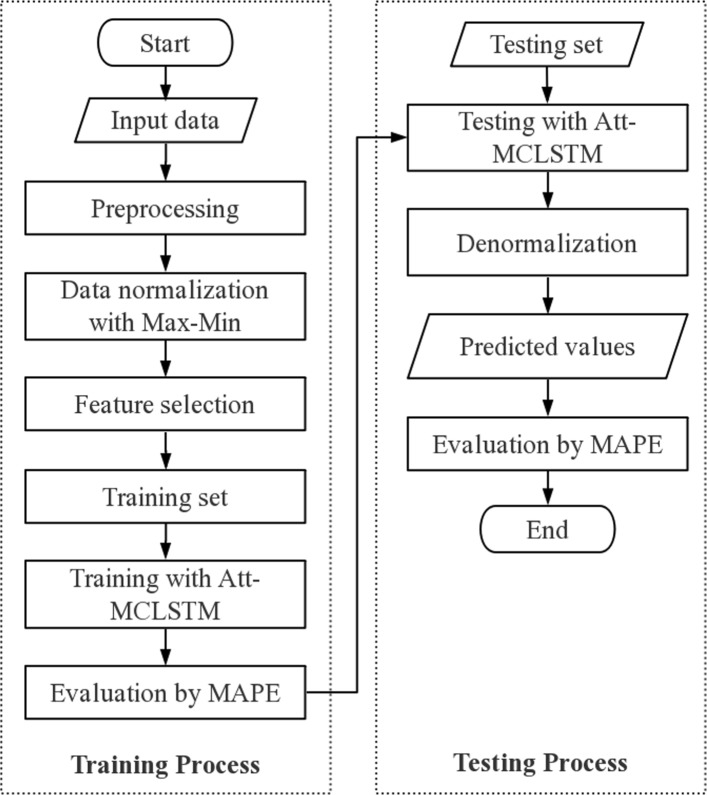
The flowchart of Attention-based multi-channel LSTM

In Fig. 1, we demonstrate the flow diagram of our method. The integrated flow diagram has two parts, training part and test part. In the training part, first, we select 19 relevant features after data cleaning and normalization processes. We further illustrate the chosen modules and features in Table 1. Table 1 doesn’t include basic information module, which includes time information, districts, and population. We use model-based ranking method as our feature selection method. In order to implement model-based ranking method, we delete one feature at a time, and input the rest of features into the same forecasting model every time. If the forecasting accuracy is low, this means that the feature we removed is relevant to our forecasting objective. After ranking all the forecasting accuracy, we select 19 features that are relevant to the forecasting objective. Then we separate the data set into training data set and test data set. The training data set contains 80 percent of data to extract annual trend and seasonal periodicity. In the test part, we test our model on the test data set. Then, we preform denormalization process to reconstruct the original values. Finally, we assess our model and compare it with other models.

**Table 1:** Modules and features description for Section 2.1

Module name	Feature name	Description
Legal influenza cases report module	Legal influenza cases numbers	The number of influenza cases in the national infectious disease reporting system.
Epidemic monitoring module	Influenza outbreaks numbers	More than 10 influenza-like cases occurred within one week in the same unit.
	Affected cases numbers	The total number of people affected by the epidemic.
Symptom monitoring module	Influenza-like cases numbers (0-5 age)	The number of influenza-like cases (0-5 age).
	Influenza-like cases numbers (5-15 age)	The number of influenza-like cases (5-15 age).
	Influenza-like cases numbers (15-25 age)	The number of influenza-like cases (15-25 age).
	Influenza-like cases numbers (25-60 age)	The number of influenza-like cases (25-60 age).
	Influenza-like cases numbers (> 60 age)	The number of influenza-like cases (over 60 age).
	Total influenza-like cases numbers	The total number of influenza-like cases.
	Total visiting patients numbers	The total number of visiting patients.
	Upper respiratory tract infections numbers	The number of upper respiratory tract infections.
Pharmacy monitoring module	Chinese patent cold medicines	Sales of Chinese patent cold medicines.
	Other cold medicines	Sales of other cold medicines.
Climate data module	Average temperature (^∘^C)	Average temperature.
	Maximum temperature (^∘^C)	Maximum temperature.
	Minimum temperature (^∘^C)	Minimum temperature.
	Rainfall (mm)	Rainfall.
	Air pressure (hPa)	Air pressure.
	Relative humidity (%)	Relative humidity.

### Data normalization

Min-Max normalization is a linear transformation strategy[27]. This method maintains the relationship among all the original data. Min-Max normalization transforms a value *x* to *y*, *y* is defined as Eq. 1.

1 $$ y = \frac{(x-min)}{(max-min)}  $$

Where *min* is the smallest value in the data, *max* is the biggest value in the data. After data normalization, the features of data will be scaled between 0 and 1.

We preform denormalization process to reconstruct the original data. Given a normalized value *y*, its original value *x* is defined as Eq. 2.

2 $$ x = (max-min)y+min  $$

### Long-short term memory neural network

Recurrent neural networks have the ability to dynamically combine experiences because of their internal recurrence[28]. Different from other traditional RNNs, LSTM can deal with the gradient vanishing problem[29]. The memory units of LSTM cells retain time series attributes of given context[29]. Some researches have proven that LSTM neural networks can yield a better performance compared with other traditional RNNs when dealing with long-term time series data[30].

**Fig. 2:**
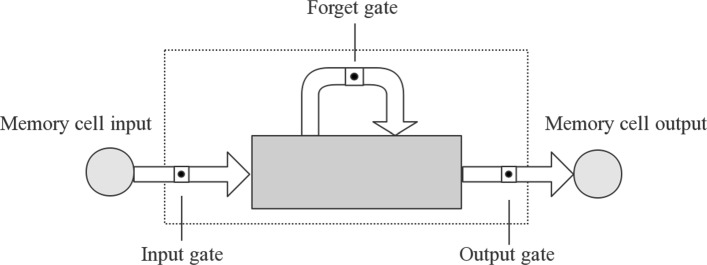
The structure of single LSTM cell

The structure of a single LSTM cell illustrate in Fig. 2. The gates control the flow of information, that is, interactions between different cells and cell itself. Input gate controls the memory state updating process. Output gate controls whether the output flow can alter other cells’ memory state. Forget gate can choose to remember or forget its previous state. LSTM is implemented by following composite functions:

3 $$\begin{array}{*{20}l} & i_{t}=\sigma(W_{xi}x_{t}+W_{hi}h_{t-1}+W_{ci}c_{t-1}+b_{i})  \end{array} $$

4 $$\begin{array}{*{20}l} & f_{t}=\sigma(W_{xf}x_{t}+W_{hf}h_{t-1}+W_{cf}c_{t-1}+b_{f})  \end{array} $$

5 $$\begin{array}{*{20}l} & c_{t}=f_{t-1}+i_{t}\tanh(W_{xc}x_{t}+W_{hc}h_{t-1}+b_{c})  \end{array} $$

6 $$\begin{array}{*{20}l} & o_{t}=\sigma(W_{xo}x_{t}+W_{ho}h_{t-1}+W_{co}c_{t}+b_{o})  \end{array} $$

7 $$\begin{array}{*{20}l} & h_{t}=o_{t}\tanh(c_{t})  \end{array} $$

Where *σ* represent the logistic sigmoid function. *i*, *f*, *o*, and *c* represent the input gate, forget gate, output gate, cell input activation vectors respectively. *h* represents the hidden vector. The weight matrix subscripts have the intuitive meaning. Like, *W*_*hi*_ represents the hidden-input gate matrix etc.

### Attention mechanism

Traditional Encode-Decode structures typically encode an input sequence into a fixed-length vector representation. However, this model has drawbacks. When the input sequence is very long, it is difficult to learn a feasible vector representation.

**Fig. 3:**
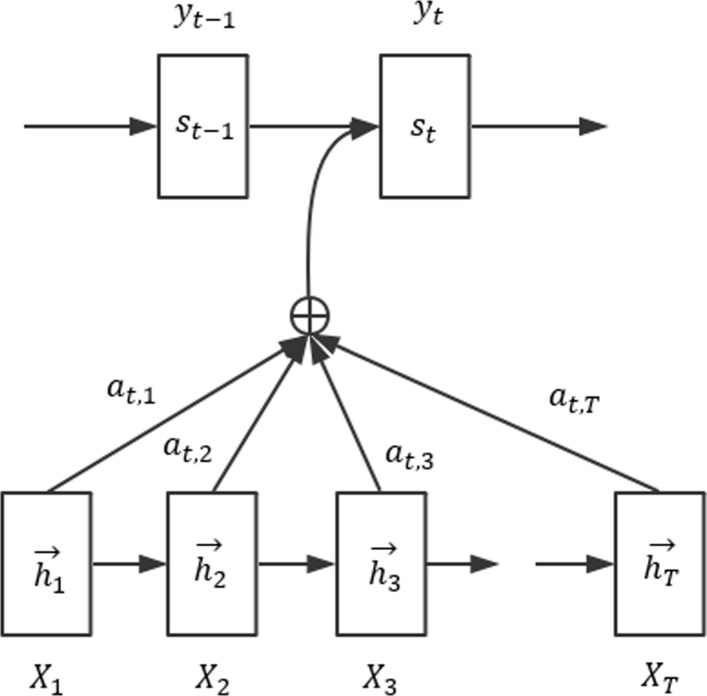
The diagram of attention mechanism. Attention layer calculates the weighted distribution of *X*_1_, …, *X*_*T*_. The input of *S*_*t*_ contains the output of the attention layer. The probability of occurrence of the output sequence …, *y*_*t*−1_, *y*_*t*_, … depends on input sequence *X*_1_, *X*_2_, …, *X*_*T*_. *h*_*i*_ represents the hidden vector. *A*_*t*,*i*_ represents the weight of *i*^*th*^ input at time step *t*

One fundamental theory of attention mechanism[31] is to abandon the conventional Encoder-Decoder structure. Attention mechanism trains a model that selectively learns the input streams by conserving the intermediate outputs of LSTM. In attention structure, the output sequences are affiliated with the input sequences. In other words, the probability of occurrence of each value in the output sequence depends on the value in the input sequence. Figure 3 illustrates the attention mechanism.

Attention layer calculates the weighted distribution of *X*_1_, …, *X*_*T*_. The input of *S*_*t*_ contains the output of the attention layer. The probability of occurrence of the output sequence …, *y*_*t*−1_,*y*_*t*_, … depends on input sequence *X*_1_, *X*_2_, …, *X*_*T*_. *h*_*i*_ represents the hidden vector. *A*_*t*,*i*_ represents the weight of *i*^*th*^ input at time step *t*. Attention layer inputs n parameters *y*_1_, …, *y*_*n*_, context sequence *c*, and outputs vector *z*, *z* is the weighted distribution of *y*_*i*_ for a given context *c*. Attention mechanism is implemented by following composite function:

8 $$\begin{array}{*{20}l} & m_{i}=\tanh(W_{cm}c+W_{ym}y_{i})  \end{array} $$

9 $$\begin{array}{*{20}l} & s_{i}\propto\exp(\langle w_{m},m_{i} \rangle)  \end{array} $$

10 $$\begin{array}{*{20}l} & \sum_{i} s_{i}=1  \end{array} $$

11 $$\begin{array}{*{20}l} & z=\sum_{i} s_{i}y_{i}  \end{array} $$

Where *m*_*i*_ is calculated by tanh layer, *s*_*i*_ is the *softmax* of the *m*_*i*_ projected on a learned direction. The output *z* is the weighted arithmetic mean of all *y*_*i*_, *W* represents the relevance for each variable according to the context *c*.

### Attention-based multi-channel LSTM

**Fig. 4:**
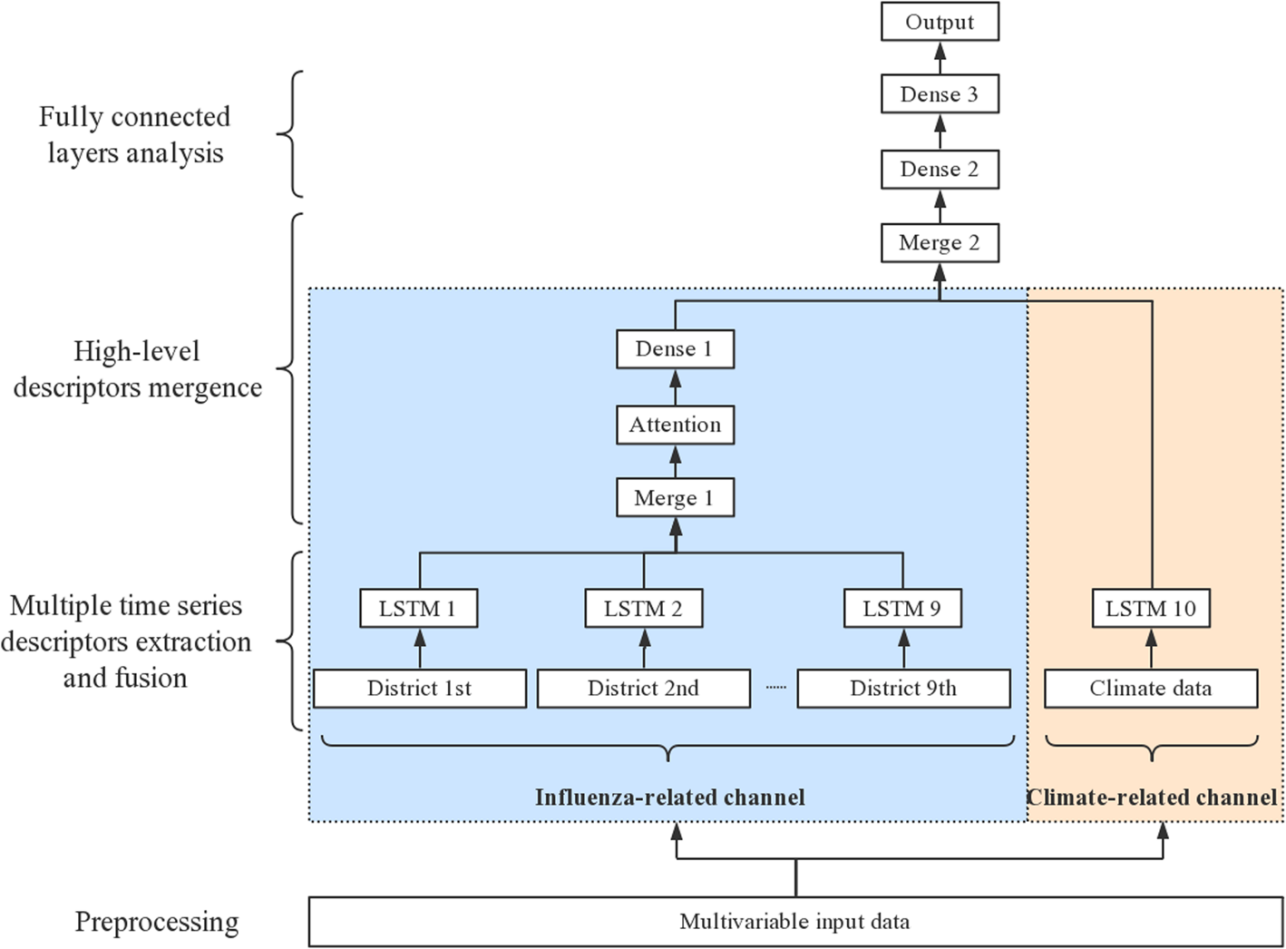
The structure of Attention-based multi-channel LSTM

In Fig. 4, we illustrate the overall architecture of our model. We separate our data set into two categories. First, we classify average temperature, maximum temperature, minimum temperature, rainfall, air pressure and relative humidity together as climate-related data category. Then, the rest of features are classified together as influenza-related data category. In our data set, each region has its own influenza-related data, and they share the same climate-related data every week.

Because our data set has the above characteristics, the inputs of Att-MCLSTM contains two parts. First, the influenza-related data is input into a series of LSTM neural networks (LSTM 1, …, LSTM 9) to capture correlative features. Second, the climate-related data is input into a single LSTM neural network (LSTM 10) to capture the long-term time series attribute of influenza epidemic data. For the first part, each LSTM neural network acquires the influenza-related data from one distinct region. In order to make full use of the complementarity among every regions, the outputs of LSTM neural networks (LSTM 1, …, LSTM 9) are concatenated in a higher layer (Merge 1). This higher layer can obtain the fused descriptors of underlying neural networks. After we capture the features of every regions, we still want to weight intermediate sequences. The reason is that the data of each region has different influences on the final forecasting result. Therefore, the intermediate sequences pass through an attention layer (Attention) and a fully connected layer (Dense 1) in turn. Thereafter, we concatenate the outputs of these two parts together (Merge 2). Finally, the intermediate sequences are passed through two fully connected layers (Dense 2, Dense 3). So far, we acquire the high-level features of the input data, and they are used to solve the influenza epidemic forecasting.

By designing a multi-channel structure, we can better extract the time-sequence property of each type of data. Not only ensures the integration of various relevant descriptors in the high-level network, but also ensures that input data will not interfere with each other in the underlying network. In the attention layer, the probability of occurrence of each value in the output sequence depends on the value in the input sequence. This structure allows us to handle the relationship of input data between different districts more appropriately.

### Evaluation method

To evaluate our method, we use the mean absolute percentage error (MAPE) as the criteria standard. Its formula is express as Eq. 12.

12 $$ MAPE = \frac{1}{n} \sum_{i=1}^{n} | \frac{y_{i}-x_{i}}{y_{i}}| \times 100  $$

Where *y*_*i*_ denotes the *i*^*th*^ actual value, and *x*_*i*_ denotes the *i*^*th*^ predicted value. If the value of MAPE is low, the accuracy of the method is high.

## Experiments

In this section, we did two experiments to verify the Att-MCLSTM model. In the first experiment, we evaluate the numbers of consecutive weeks of data that we need to forecast ILI% for the next week. In the second experiment, we compare our model with different neural network structures and other methods. Each experiment result is the average of 10 repeated trials.

### Selection of consecutive weeks

In this experiment, we set the numbers of consecutive weeks as 6, 8, 10, 12, 14 respectively. The hyper-parameters of each layer are listed in Table 2. The activation functions we used are linear activation function. The loss function and optimizer are *mape* and *adam* respectively.

**Table 2:** The size of every unit in Att-MCLSTM neural network for Section 3.1

Layer name	Units number
LSTM 1, …, LSTM 9	32
LSTM 10	32
Dense 1	16
Dense 2	10
Dense 3	1

We use the first 370 consecutive weeks’ data in training phase and the remaining data in the test phase. Each data sample includes 6 features in climate-related data category and 9 different districts’ influenza-related data. Each influenza-related data contains 13 features. The climate-related data and each district’s influenza-related data are input into the climate-related channel and the influenza-related channel respectively. The forecasting results are shown in Table 3.

**Table 3:** The MAPE of the prediction results for Section 3.1

Number of weeks	MAPE
6	0.107
8	0.092
10	0.086
12	0.106
14	0.109

### Performance validation

In this experiment, we verify the validity of our model.

- First, we compare Att-MCLSTM with MCLSTM by comparing their forecasting accuracy. The purpose of doing this is to verify the effect of the attention mechanism. For both models, we use the same multi-channel architecture (as shown in Fig.4). The only difference between these two models is that we delete the attention layer in MCLSTM. The parameters settings and data inputs method are as described in the first experiment.
- Second, we compare MCLSTM with LSTM by comparing their forecasting accuracy. The purpose of doing this is to verify the effect of the multi-channel structure. For MCLSTM, parameters settings and data inputs method are as described in the first experiment. For LSTM, we input entire features into one LSTM layer to capture the fused descriptors. Instead of separating data set according to different regions, we sum corresponding influenza-related features in each week from every regions together. Therefore, each data record includes 19 selected features. The data that contains these 19 features are passed through a fully connected layer to acquire high-level features. The units’ number of LSTM layer and fully connected layer are 32 and 1 respectively.
- Third, we demonstrate that LSTMs can yield better performance than RNNs when dealing with time series data.

## Results

(1) As can be seen from Table 3, 10 consecutive weeks’ data yields the best performance. (2) Table 4 shows that Att-MCLSTM has strong competitiveness and can provide effective real-time influenza epidemic forecasting.

**Table 4:** The MAPE of the prediction results for Section 3.2

Schemes	MAPE
Att-MCLSTM	0.086
MCLSTM	0.105
LSTM	0.118
RNN	0.132

## Discussion

The results of the first experiment indicate that 10 consecutive weeks data can appropriately reflect the time series attribute of influenza data. If the length of input data is shorter than 10, the input data doesn’t contain enough time series information. On the contrary, if the length of input data is longer than 10, the noise inside the input data increased, leading to a decrease in forecasting accuracy. Therefore, in our experiments, each data record includes 10 consecutive weeks’ data.

The results of the second experiment show that Att-MCLSTM can yield the best performance. In Table 4, from the first two rows, we can conclude that using attention mechanism can improve the MAPE from 0.105 to 0.086. The reason is that the attention layer can better deal with the relationships of input streams among every regions more appropriately. From the second row and the third row, we can conclude that using multi-channel structure can improve the MAPE from 0.118 to 0.105. The reason is that the multi-channel structure can better capture the time series attributes from different input streams. From the last two rows, we can conclude that using LSTM can improve the MAPE from 0.132 to 0.118. The reason is that LSTM neural network can better deal with time series data. This result also demonstrates the time series attribute of influenza epidemic data.

**Fig. 5:**
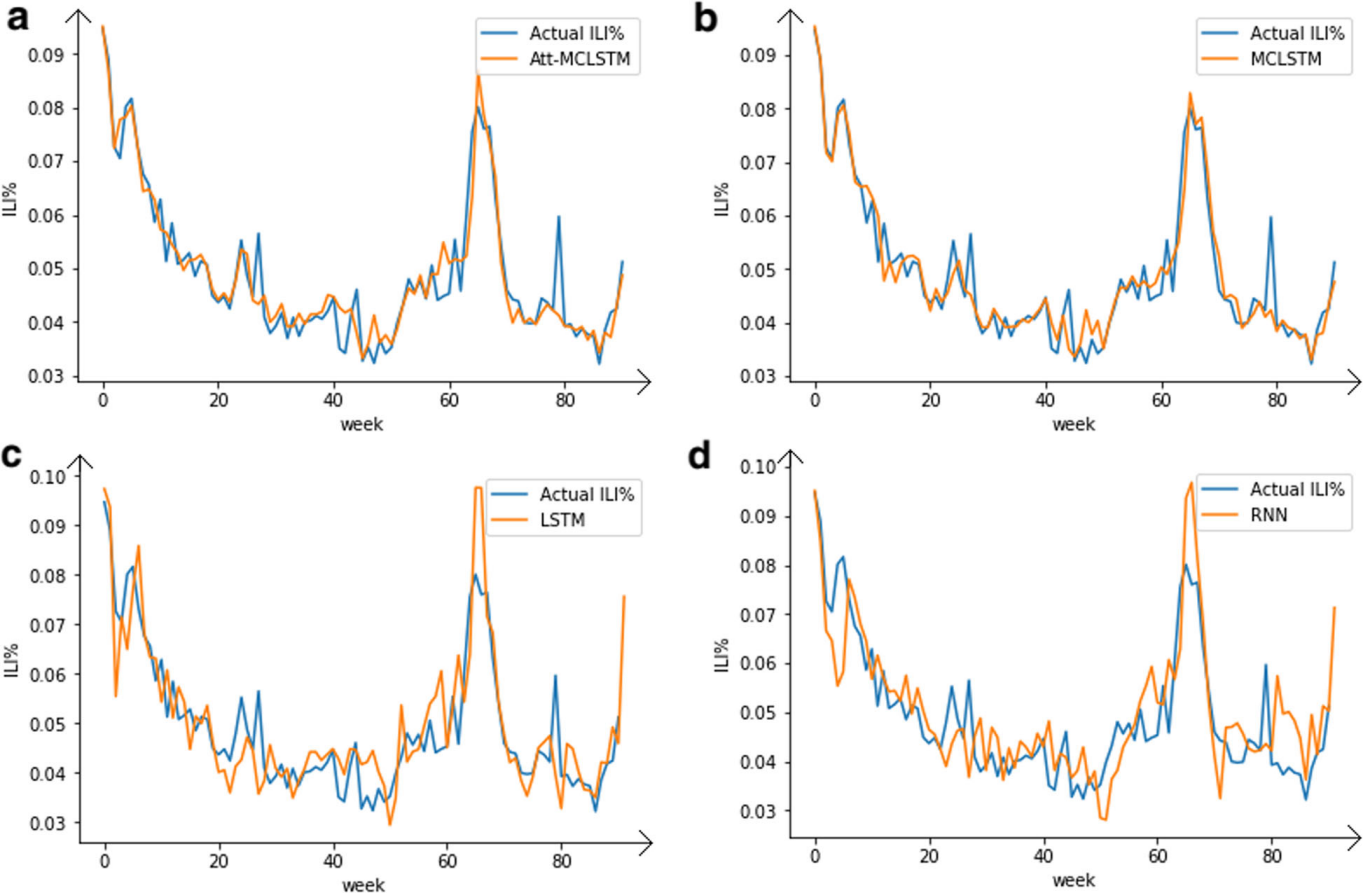
The results of one-week ahead prediction by using four individual models. **a** shows the comparison of Att-MCLSTM and real data; **b** shows the comparison of MCLSTM and real data; **c** shows the comparison of LSTM and real data; **d** shows the comparison of traditional RNN and real data. In each figure, the blue line denotes the actual values, and the orange line denotes the predicted values

Figure 5 shows the actual values and predicted values of four models. We can see that the result of Att-MCLSTM is close to the actual output. There are more obvious differences between the predicted results and the actual value by using the other three models. So, this can verify that adopting Att-MCLSTM to analyze the sequential information can help to extract time-sequence characteristic more accurately and comprehensively.

## Conclusion and future work

In this paper, we propose a new deep neural network structure (Att-MCLSTM) to forecast the ILI% in Guangzhou, China. First, we implement the multi-channel architecture to capture time series attributes from different input streams. Then, the attention mechanism is applied to weight the fused feature sequences, which allows us to deal with relationships between different input streams more appropriately. Our model fully consider the information in the data set, targetedly solving the practical problem of influenza epidemic forecasting in Guangzhou. We assess the performance of our model by comparing it with different neural network structures and other state-of-the-art models. The experimental results indicate that our model has strong competitiveness and can provide effective real-time influenza epidemic forecasting. To the best of our knowledge, this is the first study that applies LSTM neural networks to the influenza outbreaks forecasting. Continuing work will further improve the expansion ability of our model by introducing transfer learning.

## Data Availability

All data information or analyzed during this study are included in this article.

## Abbreviations

ILI: Influenza-like illness
LSTM: Long short-term memory
LASSO: Least absolute shrinkage and selection operator
MAPE: Mean absolute percentage error
